# Receptor Sorting within Endosomal Trafficking Pathway Is Facilitated by Dynamic Actin Filaments

**DOI:** 10.1371/journal.pone.0019942

**Published:** 2011-05-20

**Authors:** Emiko Ohashi, Kenji Tanabe, Yuji Henmi, Kumi Mesaki, Yuka Kobayashi, Kohji Takei

**Affiliations:** Department of Neuroscience, Okayama University Graduate School of Medicine, Dentistry and Pharmaceutical Sciences, Okayama, Japan; Iowa State University, United States of America

## Abstract

Early endosomes (EEs) are known to be a sorting station for internalized molecules destined for degradation, recycling, or other intracellular organelles. Segregation is an essential step in such sorting, but the molecular mechanism of this process remains to be elucidated. Here, we show that actin is required for efficient recycling and endosomal maturation by producing a motile force. Perturbation of actin dynamics by drugs induced a few enlarged EEs containing several degradative vacuoles and also interfered with their transporting ability. Actin repolymerization induced by washout of the drug caused the vacuoles to dissociate and individually translocate toward the perinuclear region. We further elucidated that cortactin, an actin-nucleating factor, was required for transporting contents from within EEs. Actin filaments regulated by cortactin may provide a motile force for efficient sorting within early endosomes. These data suggest that actin filaments coordinate with microtubules to mediate segregation in EEs.

## Introduction

Early endosomes (EEs) are highly dynamic compartments that act as entry portals, sorting stations, and signaling platforms [1], [2], [3]. They sort molecules and direct them into the appropriate pathway. Degradative molecules are sorted into particular membrane domains and this process is followed by maturation along with acidification and formation of intraluminar vesicles, referred to as multivesicular bodies (MVBs) [2], [4]. Finally, MVBs/late endosomes (LEs) fuse with lysosomes where protein degradation occurs. However, recycling molecules are directly transported to the plasma membrane (PM) by vesicular transport [5], [6], [7], [8] or indirectly by recycling endosomes (REs) via large tubules [9]. Much progress has been made in understanding MVB biogenesis [10]. However, the process of membrane remodeling for the recycling pathway, including tubulation and segregation activities, remains to be elucidated.

Membrane remodeling is induced by lipid-interacting proteins, lipid-modifying enzymes, and cytoskeletons and their related proteins [11], [12], [13], [14], [15], [16], [17]. Of these, recent evidence has indicated that actin plays essential roles in endosome biogenesis [18], [19], [20]. The role of actin in intracellular trafficking is well known for endocytosis, phagocytosis, and bacterial motility. In endocytosis, actin may provide a motile force to assist the fission activity of dynamin GTPase [21]. Actin functions in short-range movements through actin-rich regions [22], [23] and may be involved in endosome movement [24], cargo transport [25], [26], and endosome morphology [27]. Recent studies have shown that several actin-related proteins are required for endosomal actin reorganization. These include myosin1B [27], N-WASP [28], cortactin [26], CART, an Hrs/actinin-4/BERP/myosin V protein complex [29], Annexin A2, Spire1, and Arp2/3 [20]. As actin polymerization is a good candidate for inducing a motile force for membrane fission, understanding actin regulation of intracellular transport is sure to be a key step for further elucidating membrane trafficking.

In this report, to determine the role of actin filaments in relation to EEs, we investigated the inhibitory effects of actin dynamics on both the transport from EEs and endosome morphology. We found that inhibition of actin dynamics induced the enlargement of EEs with several distinct vacuoles and inhibited their transportation ability. Moreover, cortactin, an actin-nucleating factor, was found to be required for segregation in EEs. Thus, actin and cortactin are required for efficient transport of endosomes toward the perinuclear region. We propose that actin and cortactin play essential roles in segregation relating to the recycling and degradative pathways and in transport toward the perinuclear region in coordination with microtubules.

## Results

### Actin dynamics regulate transport beyond EEs

**Figure 1:**
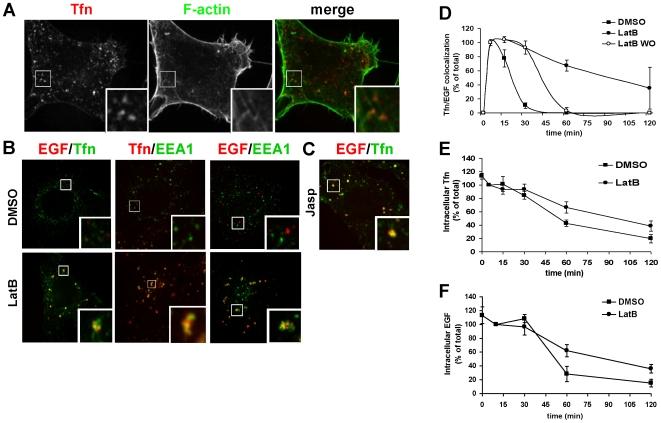
Actin localized in EEs and was required for transport from EEs. HeLa cells internalized Alexa555-Tfn for 5 min and were then fixed and stained with Alexa488-phalloidin to enable visualization of the F-actin filaments (A). Cells internalized Alexa488-EGF and Alexa555-EGF (B, left panels and C) or Alexa555-Tfn (B, center panels) and Alexa555-EGF (B, right panels) for 5 min. DMSO (B, upper panels), LatB (B, lower panels), or jasp (C) was then added to the medium and cells were incubated for 25 min. Cells were fixed and stained using anti-EEA1 (B, center and right panels; green). Signals where Tfn colocalized with EGF are summarized in D. Intracellular Tfn (E) and EGF (F) were measured using ELISA assay as described in Materials and Methods using biotin-conjugated ligands and are presented as percentages against 5 min. Error bars represent the SEM from three independent experiments.

We initially investigated whether actin plays an essential role in transport from EEs. Internalized transferrin (Tfn) signals were partially, but clearly colocalized with actin 10 min after internalization (Fig. 1A), indicating that actin localizes in EEs, as has been reported previously [24], [30]. Next, to investigate the significance of actin filaments in EEs, we used latrunculinB (LatB) as an actin depolymerizing agent [31]. For visualization of transport from EEs, we used fluorescence-labeled Tfn and EGF as tracers for the recycling and degradative pathways, respectively. HeLa cells were bound to these ligands on ice, washed, and incubated at 37°C for 5 min with ligand-free medium. Cells were then incubated with DMSO (as a control) or LatB-containing medium. At 30 min after internalization of Tfn or EGF, few Tfn signals were observed and EGF signals moved to the cell center. Both ligands were not colocalized with EEA1, an EE marker. These data indicate that Tfn was recycled and that EGF reached LEs/lysosomes (Fig. 1B, upper panels). On the other hand, in LatB-treated cells, Tfn signals were still visible and colocalized with EGF in enlarged endosomes. These enlarged endosomes had distinct vacuole domains containing EGF and also colocalized with the early endosomal marker, EEA1 (Fig. 1B, lower panels). These results suggest that intra-endosomal sorting between the degradative and recycling molecules is independent of actin, but that segregation between the pathways depends on actin polymerization. This phenotype was apparently observed only when LatB was added 5 min after ligand internalization. Following addition of LatB at 15 min after ligand internalization, the number of vacuole clusters appeared to decrease. Moreover, addition at 30 min after ligand internalization did not induce any vacuole clustering (data not shown). Next, we investigated the effect of jasplakinolide (jasp), a drug that stabilizes actin filaments and promotes actin polymerization [32]. As shown in Fig. 1C, the size and appearance of endosomes were very similar to that observed with LatB-treated cells. Although we couldn't discriminate whether these enlarged endosomes are composed from fusion or cluster of endosomes, due to limitation of resolution, these results suggest that actin dynamics including polymerization and depolymerization are required for transport from EEs.

The ratio of Tfn colocalization with EGF (Fig. 1D) in control cells showed that peak colocalization occurred at 5 min after ligand internalization and rapidly decreased until 30 min; no colocalization was observed at 60 min. In contrast, LatB treatment significantly inhibited the reduction of colocalization; however, colocalization decreased after washout of LatB, as in control cells (Fig. 1D). We further quantified the rate of recycling (Fig. 1E) and degradation (Fig. 1F) under LatB treatment using a biochemical assay. Biotin-labeled Tfn or EGF was internalized, and the intracellular contents were quantified by enzymatic activity using avidin-HRP (see Materials and Methods). In contrast to immunofluorescence data (fig. 1B), there was no significant difference between control and LatB-treated cells at 30 min after internalization. This was probably because microscopic observation largely depends on their size and concentration. At 120 min, LatB treatment significantly reduced Tfn recycling (∼40%) compared with control cells (∼20%, p<0.01, Fig. 1E). Similarly, EGF degradation was also delayed by LatB treatment (Fig. 1F). These results indicate that actin dynamics play a role in both recycling and degradation.

The recycling pathway has two independent routes; one is a direct pathway from EEs to PM and the second is an indirect pathway to PM via REs [9]. The direct pathway uses vesicle transport, which requires PI3-kinase activity and is inhibited by LY294002 (LY), a specific inhibitor of PI3-kinase [7]. To determine which pathway is dependent on actin dynamics, we used LY in combination with LatB. As shown in Figure S1A, Tfn was recycled or dissociated from EGF in control or LY-treated cells. EGF-containing endosomes were relocated to the perinuclear region, suggesting that EGF was transported to LEs/lysosomes. On the other hand, in LatB- or LatB/LY-treated cells, Tfn remained colocalized with EGF even at 30 min after internalization and these EEs were enlarged. This colocalization persisted until at least 60 min after internalization (data not shown). The quantitative analysis showed that intracellular Tfn was significantly increased in LatB/LY-treated cells (∼82%) compared with control cells (∼45%), LY- (∼62%), and LatB-treated cells (∼66%, Fig. S1B). These effects of LatB/LY on Tfn recycling suggest that the LatB-sensitive pathway differs from the LY-sensitive pathway and may be an EE-to-RE pathway.

### Disruption of actin dynamics induces enlarged endosomes

**Figure 2:**
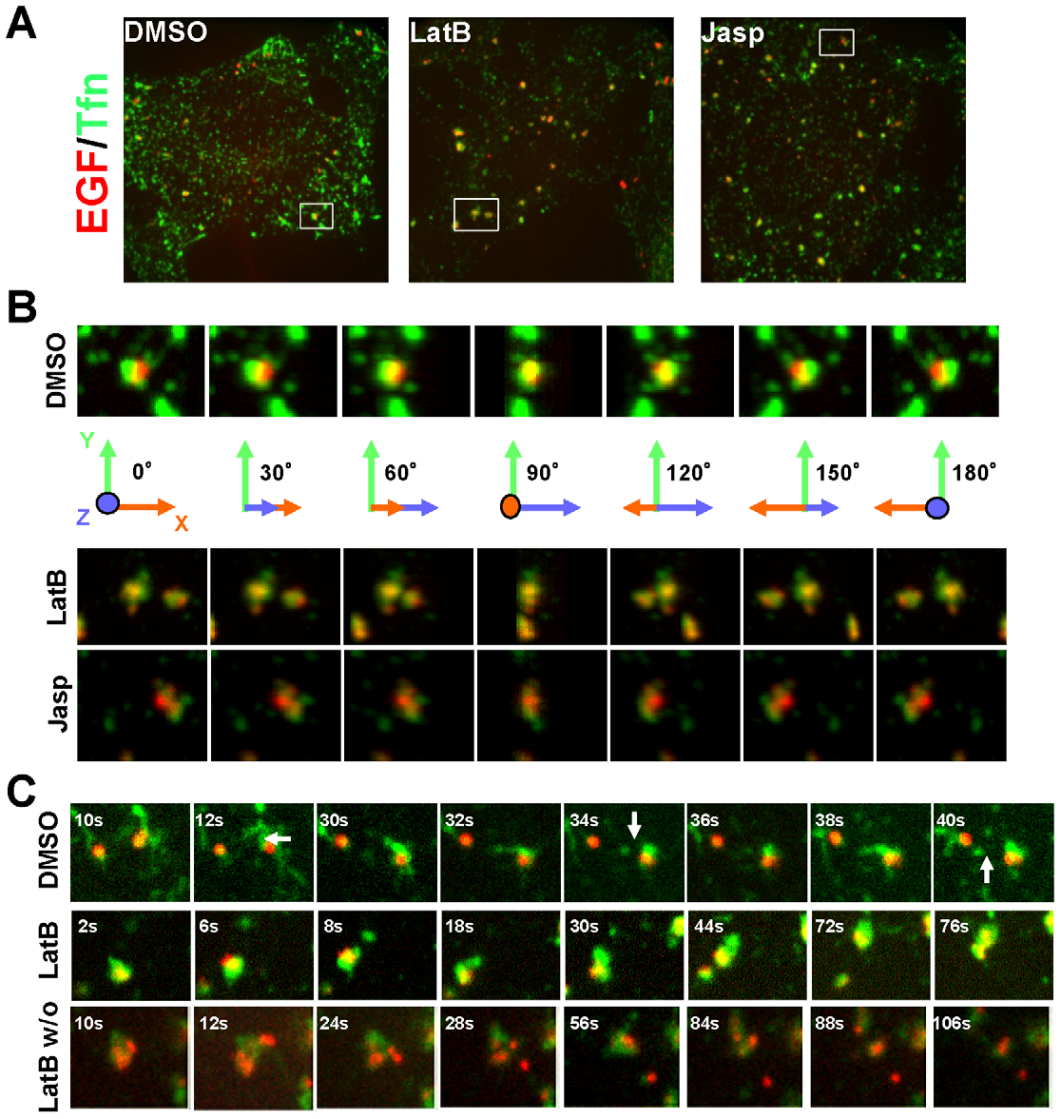
Inhibition of actin dynamics induced homotypic fusion of EEs. HeLa cells internalized both Alexa488-Tfn and Alexa555-EGF for 5 min at 37°C before the addition of DMSO (left), LatB (5 µg/ml, center), or jasp (right). Images were taken at 15 min (DMSO) or 25 min (LatB and jasp) (A). The structure of endosomes containing both EGF and Tfn was visualized by 3D reconstruction with a rotation angle of 30° (B). Live imaging of EEs. Cells were treated as in A, and images were taken at 10 min (DMSO) or 25 min (LatB) after the addition of each drug. After 30 min, LatB-treated cells were washed and images were taken at 5 min (LatB w/o). Representative images are shown. The arrow indicates a fission event.

As disruption of actin dynamics induced the formation of abnormal enlarged endosomes, we compared endosomal structure between control-, LatB-, and jasp-treated cells using 3D reconstruction (Fig. 2A). In control cells, endosomes containing both EGF and Tfn were observed until 15 min after internalization (Fig. 2B and movie S1), but not at 30 min after internalization (see Fig. 1B). On the other hand, in LatB- or jasp-treated cells, endosomes containing several vacuolar domains as well as tubular domains were seen even at 30 min after internalization (Fig. 2B, movie S2 and S3). The vacuolar domain-containing endosomes were not detected in control cells.

Next, we observed the formation of enlarged endosomes in LatB-treated cells using live cell imaging. In control cells, Tfn-containing tubules extended from endosomes and fission was observed frequently (Fig. 2C, upper panel and movie S4). However, in LatB-treated cells, endosomes rapidly fused with each other, resulting in enlarged endosomes with few short tubules (Fig. 2C, middle panel and movie S5). After washout of LatB, Tfn-containing tubular structures immediately segregated from endosomes and clusters of vacuolar domains dissociated from each other (Fig. 2C, bottom panel and movie S6). At 15 min after washout, these clusters were dissociated, and at 60 min after washout, EGF-containing endosomes localized around the perinuclear region and finally disappeared (data not shown). These data clearly indicate that disruption of the actin filaments induced aggregation of EEs, resulting in the formation of enlarged EEs. On the other hand, actin polymerization made the vacuolar domains pull apart and severed the tubules containing recycling molecules.

### Disruption of actin filaments is not required for transition to the LE and RE stage

**Figure 3:**
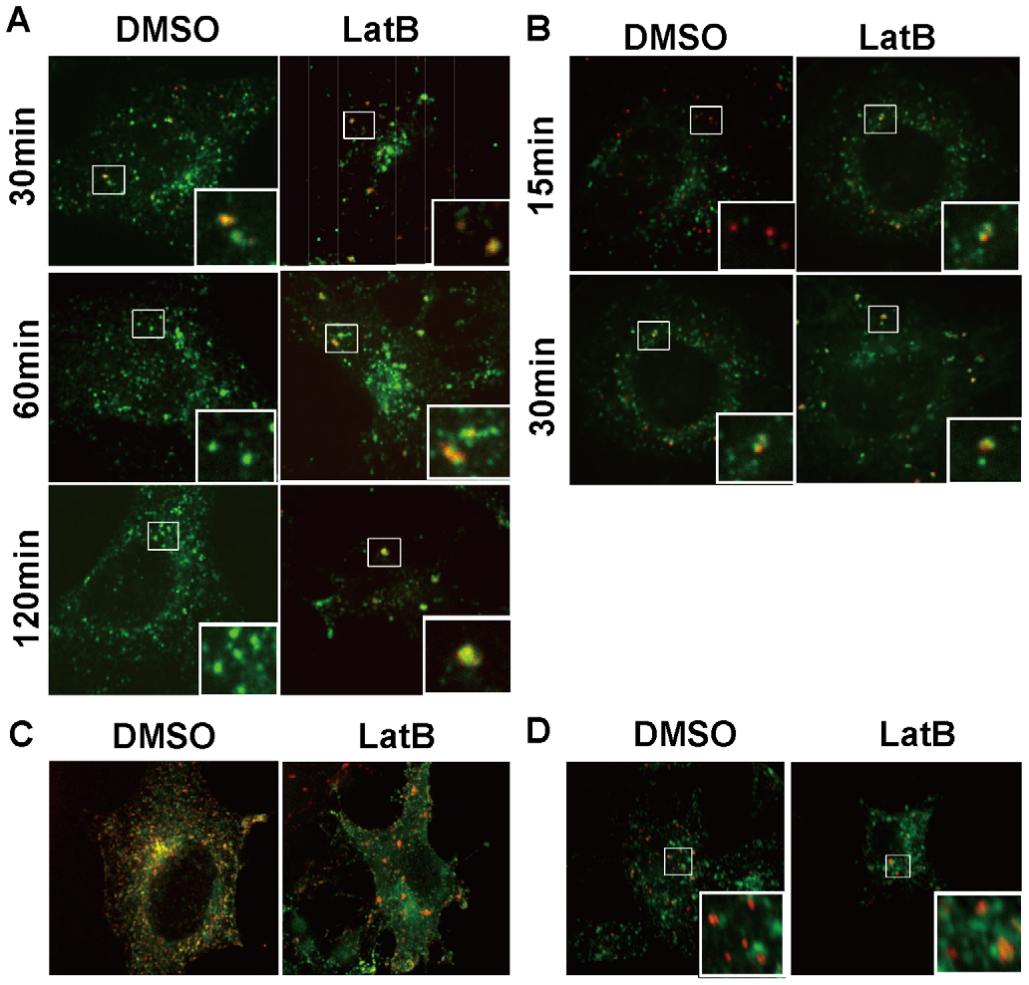
Actin depolymerization blocked transition from EE to LE. HeLa cells internalized Alexa555-EGF for 5 min at 37°C and DMSO or LatB was then added. After 30, 60, or 120 min, cells were fixed and stained with anti-Lamp1 (A) or incubated with Lysotracker (B) for 15 min or 30 min. GFP-rab11 transfected HeLa cells were internalized Alexa555-transferrin, treated with LatB for 30 min and fixed (C). Cells, which were treated with LatB for 30 min, fixed and stained with anti-EEA1 (red) and anti-Lamp1 (green. D). Samples were observed using a confocal microscope.

We demonstrated that LatB treatment induced abnormal enlargement of EEs, judging from colocalization with EEA1. However, there was a possibility that LatB treatment blocked the transition from EEs to LEs and/or REs because EEs have a mosaic structure [33]. EEs move from the cell periphery to perinuclear region in a microtubule-dependent manner and mature to LEs; this process is accompanied by both recruitment of an LE marker LAMP1 and intraluminar acidification [2], [4], [34]. Therefore, we investigated the effect of actin polymerization on endosomal maturation. In control cells, the EGF signals were colocalized with Lamp1 at 30, 60, and 120 min after internalization (Fig. 3A). Interestingly, the same results were obtained in LatB-treated cells, indicating that EEs containing EGF were partially converted to LEs. The same results were obtained using lysotracker, an acidic sensor (Fig. 3B). On the other hand, Rab11, a marker of REs, was not colocalized with EGF (Fig. 3C), suggesting that transferrin did not reach recycling endosomes. When we analyzed whether early and late endosomes fuse together in a heterotypic manner by localization of these specific markers, they were not co-localized but adjacently localized (Fig. 3D). These results indicate that the transition from EE to LE did not depend on actin dynamics, although the degradative/recycling components remain the same organelle.

### Actin contributes to early endosomal motility

**Figure 4:**
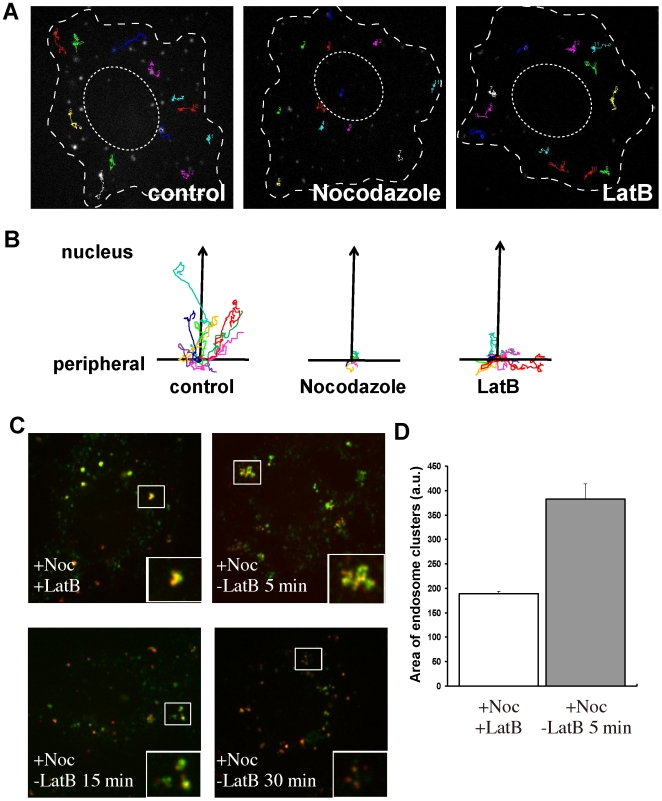
Actin pulls endosomes apart for directional movement. HeLa cells internalized Alexa555-EGF for 5 min at 37°C and DMSO or LatB was added subsequently. Nocodazole was added before the addition of the ligands and cells were incubated for 1 h at 37°C. Live cell images were taken at 15 min after internalization using confocal microscopy and a pattern of colors and dots was assigned to each particle. Shape of cells and nucleus are illustrated by dotted lines. Twelve representative tracks are shown for each treatment. The movement of EGF-positive vesicles was tracked with Image J software (A). The movement of the most motile EGF-positive endosomes was tracked relative to the center of the nucleus of each cell. Eight representative tracks (each indicated by a different color) chosen randomly from three independent experiments are shown for each treatment (B). HeLa cells internalized both Alexa488-Tfn and Alexa555-EGF for 5 min at 37°C before the addition of LatB (5 µg/ml). Fifteen minutes after the 37°C incubation, nocodazole (10 uM) was added to the mixture and this was incubated further in the presence of LatB and nocodazole. At 60 min after the addition of nocodazole, the medium was replaced with one containing only nocodazole. Cells were then further incubated for 5, 15, or 30 min (D). The EE cluster area was measured as described in Materials and Methods (E). Error bars represent the SEM from three independent experiments.

Actin filaments have been reported to be responsible for short-range movement of peripheral endosomes [35], [36]. In contrast, microtubules are responsible for long-range movements between the perinuclear and peripheral region. Therefore, we compared endosomal motility in the presence of LatB and nocodazole (a microtubule depolymerizing drug). In control cells, long-range directional movements toward the cell center were observed (Fig. 4A and movie S7). In contrast, we hardly detected any endosomal movements in nocodazole treated cells, suggesting that endosomal movements largely depend on microtubules (Fig. 4A and movie S8). However, in LatB-treated cells, EGF-containing endosomes moved rapidly in random directions and fused with each other (Fig. 4A and movie S9). Endosomes moved toward the cell center in the control cells, but in LatB-treated cells few movements toward the perinuclear region were observed despite frequent random movements (Fig. 4B). The tracking analysis clearly indicated that actin polymerization was required for directional movement toward the perinuclear region.

Next, to observe more directly the endosomal movements induced by actin polymerization, we used both LatB and nocodazole, followed by removal of only LatB to induce actin polymerization. When cells were treated with both LatB and nocodazole, enlarged EEs containing several clusters of EGF were observed (Fig. 4C). However, after removal of LatB, the EGF clusters spread quickly and had both tubular and vacuolar domains. As time progressed, EGF-containing vacuolar domains dispersed further and Tfn gradually disappeared. To quantify this dispersion, we measured the area of endosomes (Fig. 4D). The EE area was significantly increased at 5 min after the LatB washout. These results indicate that actin polymerization is required for both inhibition of homotypic fusion of endosomes through microtubule-independent movements and transport from EEs.

### Cortactin regulates actin dynamics at EEs

**Figure 5:**
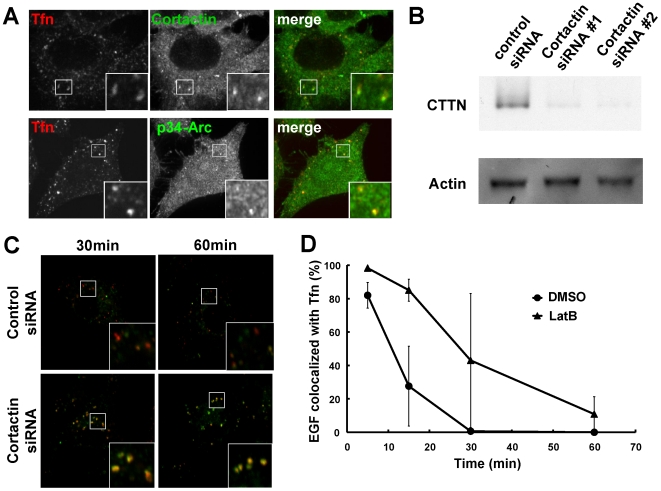
Cortactin is required for endosome biogenesis. HeLa cells internalized Alexa555-Tfn for 5 min at 37°C and were then fixed and stained with anti-cortactin (A, upper panels) or anti-p34-Arc (A, lower panels). For depletion of cortactin, negative control or cortactin-specific siRNAs were transfected into cells and these were then incubated for 72 h. Cells were lysed and processed for Western blotting using anti-cortactin or anti-actin (B). SiRNA-transfected cells internalized both Alexa488-Tfn and Alexa555-EGF for 30 or 60 min (C) or Alexa555-EGF for 5 min and LatB for 30 min. After washout of LatB, cells were incubated for 10 or 30 min and then fixed and stained using Alexa488-phalloidin to enable visualization of the F-actin filaments (D). The rate of EGF colocalized with Tfn was calculated and summarized in E.

We attempted to identify a specific regulator of actin dynamics at EEs since Arp2/3 and its regulatory elements have been suggested to play an essential role in EEs [18], [20] and its activator cortactin [37]. We also verified localization of Arp2/3 and cortactin at endosomes (Fig. 5A) and determined whether cortactin was required for endosomal sorting and recovery from LatB treatment. Cortactin was depleted by introducing cortactin-specific siRNAs (Fig. 5B), and the effects on both the recycling and degradation pathways were investigated. As shown in Fig. 5C, Tfn remained with EGF at 30 min after internalization in cortactin siRNA cells. Even at 60 min after internalization, Tfn signals were clearly recognized in cortactin siRNA cells and were partially colocalized with EGF (Fig. 5C and summarized in Fig. 5D). Similar results were obtained by another cortactin siRNA (not shown). These data suggest that segregation at EEs was impaired in cortactin-siRNA cells.

Cortactin-siRNA cells were subsequently treated with LatB and their recovery after treatment was observed. As described above, at 10 min after LatB washout, both dispersion and segregation of endosomes was observed in control cells (Fig. 5C, upper panels). On the other hand, clusters of endosomes were still observed at 30 min after washout in cortactin-siRNA cells. These results indicate that cortactin is required for actin assembly in EEs and for subsequent segregation.

## Discussion

**Figure 6:**
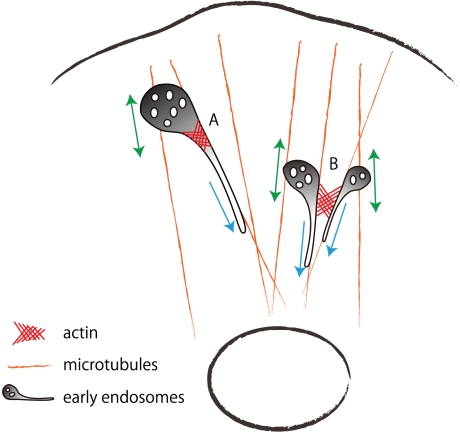
Hypothetical model of the role of actin filaments in EEs. EEs are transported on microtubules from the cell periphery to the perinuclear region. EEs are segregated into degradative vacuoles or recycling tubular endosomes. Actin filaments may regulate transport from EEs at two distinct steps. (A) In the EE-to-RE pathway, actin filaments may produce tension facilitating segregation of tubular endosomes. (B) In the EE-to-LE/lysosome pathway, actin filaments may prevent homotypic fusion of EEs, enabling each endosome to be transported from the cell periphery to the perinuclear region.

The role of actin in endosomes remains unclear, and several not mutually exclusive scenarios can be evoked. These include regulated endosome anchoring onto the actin network at the cell periphery, remodeling of the actin network by endocytic vesicles along their trajectory, endosome motility along existing actin filaments, and possible rocketing via de novo F-actin formation. Alternatively, actin may play an active role in membrane remodeling during endosome biogenesis. In this study, we revealed that actin is required for segregation in EEs, and it induces movement of each endosome toward the cell center by preventing their fusion. Further, we identified cortactin as a key regulator of actin in EEs. We propose that both actin and cortactin are involved in transport from EEs and that these function in two distinct steps (a hypothetical model is illustrated in Fig. 6).

### Actin filaments are involved in efficient segregation in EEs

The recruitment of actin filaments to EEs has been observed previously [30], [38]. Here, we demonstrated that inhibition of actin dynamics led to the formation of enlarged EEs and impaired transport from EEs. Simultaneous application of LatB and LY significantly inhibited Tfn recycling compared with individual LatB and LY applications. These results suggest that LatB and LY act in distinct pathways, and that actin might be involved in the EE-to-RE pathway, which is independent of PI3K. In fact, LatB treatment resulted in reduction in tubule formation from EEs. This leads to the major question, what is the role of actin in transport from EEs? Recently, it has been shown that actin dynamics induce scission of membrane tubules [39]. Other researchers have also suggested that actin dynamics play a role in membrane scission [21], [40]. These experiments focused on the internalization steps at PM, but this actin-induced scission may also apply to EEs. SNX4 has been shown to be a candidate factor driving membrane tubulation in the EE-to-RE pathway [41], and may contribute to membrane tubulation and scission together with actin dynamics. Another study also reported that myosin VI (a minus end-directed actin motor) and its interacting protein lemur tyrosine kinase 2 siRNAs led to swollen, enlarged EEs and reduced EHD3-containing tubule formation [19]. These results suggest that actin motor proteins also participate in the EE-to-RE pathway. Indeed, at the trans-Golgi network, GOLPH3 bridges phosphatidylinositol and actomyosin to promote efficient tubulation and vesicle formation [42]. We propose that actin filaments contribute to efficient fission by cooperating with factors driving membrane tubulation, such as SNX4 (Fig. 6A).

### Actin is required for perinuclear localization of endosomes

Actin polymerization has been shown to be involved in homotypic fusion of endosomes as well as yeast vacuoles. In *Dictyostelium*, inhibition of actin polymerization induced LEs to form clusters and blocked endosomal transport and movement, suggesting that the actin coat surrounding LEs prevents endosomes from clustering, docking, and fusing with each other. [43] On the other hand, fusion between phagosomes and LEs and between LEs themselves is affected by the inhibition of actin polymerization in vitro, indicating that the actin filaments assembled on LEs or phagosomes may facilitate endosomal fusion. These findings suggest that the actin filaments may regulate endosomal fusion at several distinct steps during intracellular transport.

We demonstrated that EEs fused with each other forming enlarged EEs after inhibition of actin dynamics. On the other hand, induction of actin-polymerization by LatB washout induced dissociation of EEs and translocation of each vacuolar domain. Furthermore, we observed that the dissociation of EEs by actin polymerization was independent of microtubules. Thus, actin filaments may provide a track for actin-based motor proteins to prevent aggregation or homotypic fusion (Fig. 6B). As the actin-dependent movement was limited among endosomes, the observation of endosomal movement under nocodazole treatment would be difficult (Fig. 4A).

### Actin polymerization is not required for the EE-to-LE transition

We found that inhibition of actin dynamics impaired EGF degradation. Although EGF-containing endosomes were EEA1-positive, they also colocalized with LAMP1, an LE marker, and exhibited intra-endosomal acidification. These results suggest that actin dynamics may be required downstream of endosomal acidification. EEs and other endocytic compartments are acidified by V-ATPase [44], [45]. V-ATPase interacts with ARNO and Arf6 in EEs and regulates the protein degradative pathway [46]. As Arf6 has been implicated in the rearrangement of the actin cytoskeleton [47], LatB might inhibit actin rearrangement downstream of Arf6.

### Cortactin regulates actin polymerization at EEs

We have shown that the Arp2/3 complex, which initiates assembly of new filaments from the sides of pre-existing filaments to generate a network of branched filament arrays, exists in EEs. In general, the Arp2/3 complex is stimulated by binding to the side of actin filaments as well as to a nucleation-promoting factor (NPF). NPFs include N-WASP, WASP, and cortactin. Cortactin has been identified as a filamentous, actin-binding protein and is a major substrate for Src kinase [48]. In addition to linking actin organization and signal transduction, cortactin has emerged as a key protein involved in the coordination of membrane dynamics and cytoskeleton remodeling. Recent evidence shows that protein kinase Cδ and calmodulin regulate receptor recycling from EEs through Arp2/3 and cortactin [26]. Our results show that cortactin may also be a key regulator of actin dynamics at EEs as cortactin siRNA impaired transport from EEs. A recent report has also revealed that WASH (an NPF) is required for endosome fission [18]. WASH localizes on Tfn-containing EEs and is required for efficient recycling from these. Cortactin may be a downstream target of WASH at EEs as WASH siRNA resulted in the loss of cortactin recruitment onto EEs. Determining the network of actin regulators and tubulation driving factors would provide precise knowledge of the sorting machinery involved in intracellular membrane transport.

## Materials and Methods

### Cells, reagents, antibodies and construct

HeLa cells were cultured in Dulbecco's modified Eagle's medium (DMEM) containing 10% fetal bovine serum (FBS) at 37°C under 5% CO_2_. LatB was purchased from Biomol, jasp from Calbiochem, nocodazole, dynasore from Sigma. Rabbit monoclonal anti-EEA1 was purchased from Cell signaling technologies. Mouse monoclonal anti-EEA1 was purchased from BD Transduction Laboratories, and mouse monoclonal anti-LAMP1 and rabbit polyclonal anti-cortactin were purchased from Santa Cruz. Rabbit polyclonal anti-p34-Arc was purchased from Upstate Biotechnology. Alexa555-conjugated Tfn, Alexa555-conjugated EGF, Alexa488-conjugated goat anti-mouse IgG, anti-rabbit IgG, Alexa488-conjugated phalloidin, and Lysotracker DND-26 were purchased from Invitrogen. GFP-Rab11 was a generous gift from M. Fukuda (Tohoku Univ., Japan).

### RNA interference and plasmid transfection

Cortactin siRNAs (siRNA CTTN-1: 5′-CCGAAUGGAUAAGUCAGCUtt-3′, siRNA CTTN-2: 5′-GGUUUCGGCGGCAAAUACGtt-3′), N-WASP siRNAs (siRNA WASL-1: 5′-GCAGAUCGGAACUGUAGUGtt-3′, siRNA WASL-2: 5′-GGGUAUCCAACUAAAAUCUtt-3′, siRNA WASL-3: 5′-GGUAAUAUUAUGGUAACUCtt-3′), and negative control siRNA were purchased from Ambion. HeLa cells plated in 35-mm diameter dishes were transfected with each duplex siRNA (100 pmol) using Lipofectamin2000 (Invitrogen), according to the manufacturer's instructions. The cells were used in the different experiments after 72 h of incubation. To observe recycling endosomes, GFP-rab11 was transfected by Effectene (QIAGEN) and analyzed at 24 h after transfection.

### Immunofluorescence

HeLa cells cultured on coverslips were starved with serum-free medium containing 0.1% BSA/DMEM for 1 h. For Tfn and EGF binding, the medium was replaced with serum-free DMEM containing Alexa488- or Alexa555-conjugated Tfn or Alexa555-conjugated EGF (Molecular Probes) and cells were incubated for 1 h on ice. Cells were then washed with cold phosphate-buffered saline and incubated with 10% FBS/DMEM at 37°C. LatB (5 µg/ml), Jasp (1 µM) or LY294002 (100 µM) were added 5 min after Tfn and EGF internalization and cells were incubated for the time periods described. The treated cells were fixed with 3.7% formaldehyde for 15 min at RT and immunofluorescence was performed as described previously [49].

### Imaging analysis

Colocalization of Tfn and EGF fluorescence at EEs was calculated using MetaMorph software (Universal Imaging Corporation). The threshold of fluorescence was determined by a 15 min-based DMSO control. Colocalization was calculated by measuring the ratio of Tfn to EGF signals. We quantified 10 cell images and averaged these (excluding two extreme values). For 3D reconstruction, Z-series imaging of the samples was performed in 0.1-µm increments. These images were reconstructed with MetaMorph software. For area measurements, the endosome cluster area was enclosed with a circle and measured with Image J software (National Institutes of Health).

### Live imaging and tracking analysis

HeLa cells plated on a 35-mm diameter dish with a glass base were treated with DMSO or LatB as in the immunofluorescence experiments. Time-lapse images were taken using a confocal microscope and were acquired every 2 s. These images were reconstituted with MetaMorph software. The tracking analysis samples were taken using video microscopy, and the tracking analysis was performed with Image J software (National Institutes of Health).

### ELISA assay

HeLa cells were plated on a 24-well plate, grown in serum-free medium for 1 h, and incubated with biotin-human transferrin or biotin-human EGF for 1 h on ice to facilitate binding to the cell surface. The ligands were internalized at 37°C, and cells were incubated for the times indicated with the appropriate reagents. To measure Tfn recycling and EGF degradation, ELISA assays were performed as described [50]. A peroxidase coloring kit (SUMILON) was used for the enzymatic reaction and light absorption was determined with a microplate reader (MTP 300; Hitachi) in order to quantify intracellular Tfn and EGF.

## Supporting Information

Figure S1 **Actin polymerization participates in EE-to-RE transport.** HeLa cells internalized both Alexa488-Tfn and Alexa555-EGF for 5 min before the addition of DMSO, LY294002 (LY), and LatB or both LY and LatB. Cells were then incubated for further 30 min, fixed, and observed (A). Intracellular Tfn was measured as in Fig. 1E (B). Error bars represent the SEM from three independent experiments performed in duplicate.
(TIF)

Movie S1 **3D reconstitution of early endosomes containing both Tfn and EGF.** HeLa cells internalized both Alexa488-Tfn and Alexa555-EGF for 5 min at 37°C before the addition of DMSO. Images were taken at 15 min (DMSO). The structure of endosomes containing both EGF and Tfn was visualized by 3D reconstruction with a rotation angle of 2°. Frame rate, 7 frames/sec (181 frames).
(AVI)

Movie S2 **3D reconstitution of early endosomes under LatB treatment.** HeLa cells internalized both Alexa488-Tfn and Alexa555-EGF for 5 min at 37°C before the addition of LatB. Images were taken at 25 min. The structure of endosomes containing both EGF and Tfn was visualized by 3D reconstruction with a rotation angle of 2°. Frame rate, 7 frames/sec (181 frames).
(AVI)

Movie S3 **3D reconstitution of early endosomes under Jasplakinolide treatment.** HeLa cells internalized both Alexa488-Tfn and Alexa555-EGF for 5 min at 37°C before the addition of Jasp. Images were taken at 25 min. The structure of endosomes containing both EGF and Tfn was visualized by 3D reconstruction with a rotation angle of 2°. Frame rate, 7 frames/sec (181 frames).
(AVI)

Movie S4 **Tubular endosomes was segregated from early endosomes.** HeLa cells internalized both Alexa488-Tfn and Alexa555-EGF for 5 min at 37°C before the addition of DMSO, and images were taken at 10 min after the addition of DMSO. Images were collected every 2 sec for 210 sec. Frame rate, 7 frames/sec (105 frames).
(AVI)

Movie S5 **Large early endosomes were formed by clustering of several early endosomes under LatB treatment.** HeLa cells internalized both Alexa488-Tfn and Alexa555-EGF for 5 min at 37°C before the addition of LatB, and images were taken at 25 min after the addition of the drug. Images were collected every 2 sec for 2 min. Frame rate, 7 frames/sec (60 frames).
(AVI)

Movie S6 **Actin polymerization induced by removal of LatB made extension and fission of tubular endosomes.** HeLa cells internalized both Alexa488-Tfn and Alexa555-EGF for 5 min at 37°C before the addition of LatB. After 30 min, LatB-treated cells were washed and images were taken at 5 min. Images were collected every 2 sec for 108 sec. Frame rate, 7 frames/sec (54 frames).
(AVI)

Movie S7 **Directional movements of EGF-containing endosomes.** HeLa cells internalized Alexa555-EGF for 5 min at 37°C, and DMSO was added subsequently. Live cell images were taken at 15 min after internalization using confocal microscopy. Images were collected every 2 sec for 3 min, and a pattern of colors and dots was assigned to each particle. Frame rate, 7 frames/sec (90 frames).
(AVI)

Movie S8 **Microtubules were required for movements of EGF-containing endosomes.** HeLa cells internalized Alexa555-EGF for 5 min at 37°C. Nocodazole was added before the addition of the ligands and cells were incubated for 1 h at 37°C. Live cell images were taken at 15 min after internalization using confocal microscopy. Images were collected every 2 sec for 3 min, and a pattern of colors and dots was assigned to each particle. Frame rate, 7 frames/sec (90 frames).
(AVI)

Movie S9 **Actin were required for directional movements of EGF-containing endosomes.** HeLa cells internalized Alexa555-EGF for 5 min at 37°C, and LatB was added subsequently. Live cell images were taken at 15 min after internalization using confocal microscopy. Images were collected every 2 sec for 3 min, and a pattern of colors and dots was assigned to each particle. Frame rate, 7 frames/sec (90 frames).
(AVI)

## References

[1] Mellman I (1996). Endocytosis and molecular sorting.. Annu Rev Cell Dev Biol.

[2] Futter CE, Pearse A, Hewlett LJ, Hopkins CR (1996). Multivesicular endosomes containing internalized EGF-EGF receptor
complexes mature and then fuse directly with lysosomes.. J Cell Biol.

[3] Sadowski L, Pilecka I, Miaczynska M (2009). Signaling from endosomes: location makes a
difference.. Exp Cell Res.

[4] Stoorvogel W, Strous GJ, Geuze HJ, Oorschot V, Schwartz AL (1991). Late endosomes derive from early endosomes by
maturation.. Cell.

[5] Hao M, Maxfield FR (2000). Characterization of rapid membrane internalization and
recycling.. J Biol Chem.

[6] Sheff DR, Pelletier L, O'Connell CB, Warren G, Mellman I (2002). Transferrin receptor recycling in the absence of perinuclear
recycling endosomes.. J Cell Biol.

[7] van Dam EM, Ten Broeke T, Jansen K, Spijkers P, Stoorvogel W (2002). Endocytosed transferrin receptors recycle via distinct dynamin
and phosphatidylinositol 3-kinase-dependent pathways.. J Biol Chem.

[8] van Dam EM, Stoorvogel W (2002). Dynamin-dependent transferrin receptor recycling by
endosome-derived clathrin-coated vesicles.. Mol Biol Cell.

[9] Maxfield FR, McGraw TE (2004). Endocytic recycling.. Nat Rev Mol Cell Biol.

[10] Piper RC, Katzmann DJ (2007). Biogenesis and function of multivesicular bodies.. Annu Rev Cell Dev Biol.

[11] Frost A, Unger VM, De Camilli P (2009). The BAR domain superfamily: membrane-molding
macromolecules.. Cell.

[12] Bashkirov PV, Akimov SA, Evseev AI, Schmid SL, Zimmerberg J (2008). GTPase cycle of dynamin is coupled to membrane squeeze and
release, leading to spontaneous fission.. Cell.

[13] Lanzetti L (2007). Actin in membrane trafficking.. Curr Opin Cell Biol.

[14] Yarar D, Waterman-Storer CM, Schmid SL (2007). SNX9 couples actin assembly to phosphoinositide signals and is
required for membrane remodeling during endocytosis.. Dev Cell.

[15] Shinozaki-Narikawa N, Kodama T, Shibasaki Y (2006). Cooperation of phosphoinositides and BAR domain proteins in
endosomal tubulation.. Traffic.

[16] Antonny B (2006). Membrane deformation by protein coats.. Curr Opin Cell Biol.

[17] Bard F, Malhotra V (2006). The formation of TGN-to-plasma-membrane transport
carriers.. Annu Rev Cell Dev Biol.

[18] Derivery E, Sousa C, Gautier J, Lombard B, Loew D (2009). The Arp2/3 Activator WASH Controls the Fission of Endosomes
through a Large Multiprotein Complex.. Dev Cell.

[19] Chibalina MV, Seaman MNJ, Miller CC, Kendrick-Jones J, Buss F (2007). Myosin VI and its interacting protein LMTK2 regulate tubule
formation and transport to the endocytic recycling
compartment.. J Cell Sci.

[20] Morel E, Parton RG, Gruenberg J (2009). Annexin A2-dependent polymerization of actin mediates endosome
biogenesis.. Dev Cell.

[21] Roux A, Uyhazi K, Frost A, De Camilli P (2006). GTP-dependent twisting of dynamin implicates constriction and
tension in membrane fission.. Nature.

[22] Brown B, Song W (2001). The actin cytoskeleton is required for the trafficking of the b
cell antigen receptor to the late endosomes.. Traffic.

[23] Aschenbrenner L, Naccache SN, Hasson T (2004). Uncoated endocytic vesicles require the unconventional myosin,
Myo6, for rapid transport through actin barriers.. Mol Biol Cell.

[24] Taunton J, Rowning BA, Coughlin ML, Wu M, Moon RT (2000). Actin-dependent propulsion of endosomes and lysosomes by
recruitment of N-WASP.. J Cell Biol.

[25] Sheff DR, Kroschewski R, Mellman I (2002). Actin dependence of polarized receptor recycling in Madin-Darby
canine kidney cell endosomes.. Mol Biol Cell.

[26] Lladó A, Timpson P, Muga SVd, Moretó J, Pol A (2008). Protein Kinase C{delta} and Calmodulin Regulate Epidermal Growth
Factor Receptor Recycling from Early Endosomes through Arp2/3 Complex and
Cortactin.. Mol Biol Cell.

[27] Salas-Cortes L, Ye F, Tenza D, Wilhelm C, Theos A (2005). Myosin Ib modulates the morphology and the protein transport
within multi-vesicular sorting endosomes.. J Cell Sci.

[28] Chang FS, Stefan CJ, Blumer KJ (2003). A WASp homolog powers actin polymerization-dependent motility of
endosomes in vivo.. Curr Biol.

[29] Yan Q, Sun W, Kujala P, Lotfi Y, Vida TA (2005). CART: an Hrs/actinin-4/BERP/myosin V protein complex required for
efficient receptor recycling.. Mol Biol Cell.

[30] Gauthier NC, Monzo P, Gonzalez T, Doye A, Oldani A (2007). Early endosomes associated with dynamic F-actin structures are
required for late trafficking of H. pylori VacA toxin.. J Cell Biol.

[31] Spector I, Shochet NR, Kashman Y, Groweiss A (1983). Latrunculins: novel marine toxins that disrupt microfilament
organization in cultured cells.. Science.

[32] Bubb MR, Senderowicz AM, Sausville EA, Duncan KL, Korn ED (1994). Jasplakinolide, a cytotoxic natural product, induces actin
polymerization and competitively inhibits the binding of phalloidin to
F-actin.. J Biol Chem.

[33] Miaczynska M, Zerial M (2002). Mosaic organization of the endocytic pathway.. Exp Cell Res.

[34] Driskell OJ, Mironov A, Allan VJ, Woodman PG (2007). Dynein is required for receptor sorting and the morphogenesis of
early endosomes.. Nat Cell Biol.

[35] Semenova I, Burakov A, Berardone N, Zaliapin I, Slepchenko B (2008). Actin dynamics is essential for Myosin-based transport of
membrane organelles.. Curr Biol.

[36] Cordonnier MN, Dauzonne D, Louvard D, Coudrier E (2001). Actin filaments and myosin I alpha cooperate with microtubules
for the movement of lysosomes.. Mol Biol Cell.

[37] Kaksonen M, Peng HB, Rauvala H (2000). Association of cortactin with dynamic actin in lamellipodia and
on endosomal vesicles.. J Cell Sci.

[38] Lladó A, Tebar F, Calvo M, Moretó J, Sorkin A (2004). Protein kinaseCdelta-calmodulin crosstalk regulates epidermal
growth factor receptor exit from early endosomes.. Mol Biol Cell.

[39] Römer W, Pontani L-L, Sorre B, Rentero C, Berland L (2010). Actin Dynamics Drive Membrane Reorganization and Scission in
Clathrin-Independent Endocytosis.. Cell.

[40] Ferguson S, Raimondi A, Paradise S, Shen H, Mesaki K (2009). Coordinated actions of actin and BAR proteins upstream of dynamin
at endocytic clathrin-coated pits.. Dev Cell.

[41] Traer CJ, Rutherford AC, Palmer KJ, Wassmer T, Oakley J (2007). SNX4 coordinates endosomal sorting of TfnR with dynein-mediated
transport into the endocytic recycling compartment.. Nat Cell Biol.

[42] Dippold HC, Ng MM, Farber-Katz SE, Lee S-K, Kerr ML (2009). GOLPH3 bridges phosphatidylinositol-4- phosphate and actomyosin
to stretch and shape the Golgi to promote budding.. Cell.

[43] Drengk A, Fritsch J, Schmauch C, Rühling H, Maniak M (2003). A coat of filamentous actin prevents clustering of late-endosomal
vacuoles in vivo.. Curr Biol.

[44] Nishi T, Forgac M (2002). The vacuolar (H+)-ATPases–nature's most versatile
proton pumps.. Nat Rev Mol Cell Biol.

[45] Sun-Wada GH, Wada Y, Futai M (2004). Diverse and essential roles of mammalian vacuolar-type proton
pump ATPase: toward the physiological understanding of inside acidic
compartments.. Biochim Biophys Acta.

[46] Hurtado-Lorenzo A, Skinner M, El Annan J, Futai M, Sun-Wada G-H (2006). V-ATPase interacts with ARNO and Arf6 in early endosomes and
regulates the protein degradative pathway.. Nat Cell Biol.

[47] Donaldson JG (2003). Multiple roles for Arf6: sorting, structuring, and signaling at
the plasma membrane.. J Biol Chem.

[48] Schafer DA (2002). Coupling actin dynamics and membrane dynamics during
endocytosis.. Curr Opin Cell Biol.

[49] Tanabe K, Torii T, Natsume W, Braesch-Andersen S, Watanabe T (2005). A novel GTPase-activating protein for ARF6 directly interacts
with clathrin and regulates clathrin-dependent endocytosis.. Mol Biol Cell.

[50] Smythe E, Redelmeier TE, Schmid SL (1992). Receptor-mediated endocytosis in semiintact
cells.. Meth Enzymol.

