# Supplementary figures and images for: Receptor Sorting within Endosomal Trafficking Pathway Is Facilitated by Dynamic Actin Filaments
Source: PLoS One. 2011 May 20;6(5):e19942. doi: 10.1371/journal.pone.0019942 (PMC3098849; doi:10.1371/journal.pone.0019942)

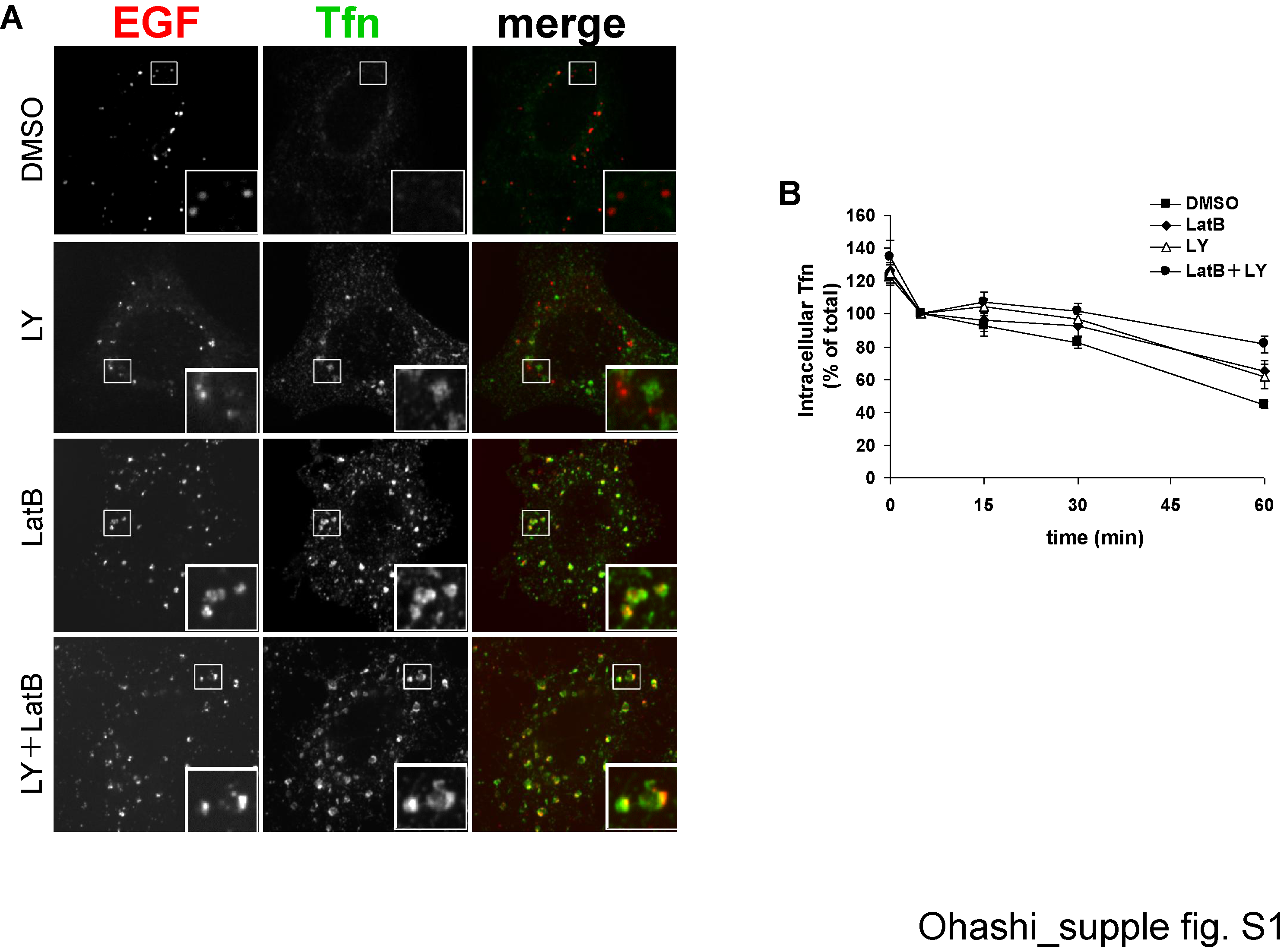

Supplement: Figure S1 — Actin polymerization participates in EE-to-RE transport. HeLa cells internalized both Alexa488-Tfn and Alexa555-EGF for 5 min before the addition of DMSO, LY294002 (LY), and LatB or both LY and LatB. Cells were then incubated for further 30 min, fixed, and observed (A). Intracellular Tfn was measured as in Fig. 1E (B). Error bars represent the SEM from three independent experiments performed in duplicate. (TIF) [file pone.0019942.s001.tif]
